# Zebrafish *enpp1* mutants exhibit pathological mineralization, mimicking features of generalized arterial calcification of infancy (GACI) and pseudoxanthoma elasticum (PXE)

**DOI:** 10.1242/dmm.015693

**Published:** 2014-06-06

**Authors:** Alexander Apschner, Leonie F. A. Huitema, Bas Ponsioen, Josi Peterson-Maduro, Stefan Schulte-Merker

**Affiliations:** 1 Hubrecht Institute – KNAW & UMC Utrecht, 3548CT Utrecht, The Netherlands.; 2 WUR, Experimental Zoology, 3700AH Wageningen, The Netherlands.; 3 Institute of Cardiovascular Organogenesis and Regeneration, Faculty of Medicine, University of Münster, 48149 Münster, Germany.

**Keywords:** Zebrafish, Ectopic mineralization, Generalized arterial calcification of infancy, GACI, Pseudoxanthoma elasticum, PXE, Pyrophosphate

## Abstract

In recent years it has become clear that, mechanistically, biomineralization is a process that has to be actively inhibited as a default state. This inhibition must be released in a rigidly controlled manner in order for mineralization to occur in skeletal elements and teeth. A central aspect of this concept is the tightly controlled balance between phosphate, a constituent of the biomineral hydroxyapatite, and pyrophosphate, a physiochemical inhibitor of mineralization. Here, we provide a detailed analysis of a zebrafish mutant, *dragonfish* (*dgf*), which is mutant for ectonucleoside pyrophosphatase/phosphodiesterase 1 (Enpp1), a protein that is crucial for supplying extracellular pyrophosphate. Generalized arterial calcification of infancy (GACI) is a fatal human disease, and the majority of cases are thought to be caused by mutations in ENPP1. Furthermore, some cases of pseudoxanthoma elasticum (PXE) have recently been linked to ENPP1. Similar to humans, we show here that zebrafish *enpp1* mutants can develop ectopic calcifications in a variety of soft tissues – most notably in the skin, cartilage elements, the heart, intracranial space and the notochord sheet. Using transgenic reporter lines, we demonstrate that ectopic mineralizations in these tissues occur independently of the expression of typical osteoblast or cartilage markers. Intriguingly, we detect cells expressing the osteoclast markers Trap and CathepsinK at sites of ectopic calcification at time points when osteoclasts are not yet present in wild-type siblings. Treatment with the bisphosphonate etidronate rescues aspects of the *dgf* phenotype, and we detected deregulated expression of genes that are involved in phosphate homeostasis and mineralization, such as *fgf23*, *npt2a*, *entpd5* and *spp1* (also known as *osteopontin*). Employing a UAS-GalFF approach, we show that forced expression of *enpp1* in blood vessels or the floorplate of mutant embryos is sufficient to rescue the notochord mineralization phenotype. This indicates that *enpp1* can exert its function in tissues that are remote from its site of expression.

## INTRODUCTION

Calcium and phosphate are the main elements in hydroxyapatite, the mineral that constitutes the vertebral skeleton and teeth. Hydroxyapatite also occurs in the form of ectopic calcifications, which can result from disease, injury or aging in a wide variety of organs and tissues. Ectopic calcifications are also often a result of imbalanced ion levels, again, specifically calcium and phosphate (in chronic kidney disease, for example) (Giachelli, 1999). Particularly when occurring in vascular tissues, ectopic calcification has been associated with increased mortality (Goodman et al., 2000; Ganesh et al., 2001).

Two key concepts have emerged from human genetic studies and animal experimental data on the control of biomineralization over the past few years. First, calcium and phosphate, which readily form an insoluble precipitate, are present in virtually all tissues and body fluids; therefore, crystallization has to be actively inhibited. Second, the balance between phosphate and pyrophosphate is a crucial determinant in the regulation of this crystallization process (Giachelli, 2008; Kirsch, 2012). Phosphate is an element that enables the formation of hydroxyapatite, whereas pyrophosphate is a strong chemical inhibitor of crystal formation (Terkeltaub, 2001).

Generalized arterial calcification of infancy (GACI; OMIM 208000) is an autosomal-recessive disorder that is characterized by the calcification of medium and large arteries in humans. It often leads to demise because of arterial stenosis and, consequently, heart failure within the first months of life. Mutations in ectonucleotide pyrophosphatase/phosphodiesterase-1 (ENPP1; formerly known as PC-1) have been identified as being causative in the majority of GACI cases investigated (Rutsch et al., 2003; Nitschke et al., 2012). Mouse models and *in vitro* data have confirmed that ENPP1 function is crucial in the regulation of biomineralization (Johnson et al., 2003; Mackenzie et al., 2012a) because ENPP1 generates extracellular pyrophosphate through the hydrolysis of extracellular ATP (Kato et al., 2012). Recently it has become clear that the spectrum of human phenotypes that are caused by mutations in ENPP1 is variable, and less severe cases present themselves with symptoms of hypophosphatemic rickets or pseudoxanthoma elasticum (PXE; OMIM 264800). PXE is predominantly characterized by mineralization in the skin and eye, as well as the vasculature, although it has a later onset than GACI (Li et al., 2012; Nitschke et al., 2012). Most cases of PXE have been associated with mutations in ABCC6 and not ENPP1; however, recently a mechanistic link between ABCC6 mutations and reduced amounts of pyrophosphate has been established (Jansen et al., 2013).

Zebrafish share many of the basic features of chondrogenesis and osteogenesis with higher vertebrates (Apschner et al., 2011; Mackay et al., 2013), and offer the opportunity to perform genetic and chemical screens (Spoorendonk et al., 2010), as well as to examine osteoblasts and osteoclasts in an *in vivo* setting. We have recently described the catalytic activity of Ectonucleoside triphosphate diphosphohydrolase 5 (Entpd5) as an essential provider of phosphate for mineralization of the zebrafish skeleton (Huitema et al., 2012). Here, we provide a detailed analysis of the *dragonfish* (*dgf*) mutant, which displays features that are found in both GACI and PXE. Furthermore, we demonstrate the suitability of the *dgf* mutant for chemical screening of drugs that inhibit mineralization and provide evidence that Enpp1 can act at areas that are distal from its site of expression. Finally, we show that ectopic mineralizations can lead to the generation of osteoclasts, a finding that has possible consequences for the treatment of GACI.

**TRANSLATIONAL IMPACT**

**Clinical issue**

Ectopic calcifications (abnormal deposition of calcium) can occur in most soft tissues of the human body. They commonly occur in individuals with uremia who experience systemic mineral imbalance; however, they are also seen in certain rare autosomal recessive disorders, such as generalized arterial calcification of infancy (GACI) or pseudoxanthoma elasticum (PXE). Although individuals with GACI develop severe calcifications in arteries and often die during the first months of life, progression in PXE is usually slower, and mineralization is more prevalent in the skin and eyes than in the vasculature. In the majority of GACI cases and some PXE cases, mutations in *ENPP1* have been implicated as the cause of the disease. ENPP1 is an ectoenzyme that metabolizes extracellular ATP to generate pyrophosphate, a chemical inhibitor of mineralization. This study sought to explore the pathological effects of *ENPP1* mutation in detail by using zebrafish.

**Results**

The recently described zebrafish *dragonfish* (*dgf*) mutant carries a mutation in *enpp1*. The present study reveals that the *dragonfish* mutant displays canonical features of human GACI and PXE, such as mineralization in the blood vasculature, skin and eye. The authors show that ectopic calcifications in *dgf* mutants appear independently of osteogenic cell marker expression, suggesting that calcifications are a result of passive calcium deposition. Furthermore, they show that treatment with etidronate, a bisphosphonate that is used to treat human GACI, is able to alleviate ectopic calcifications. This confirms that decreased pyrophosphate levels underlie the phenotype in *dgf* mutants, which is supported by the detection of deregulated expression of genes involved in phosphate homeostasis and mineralization. The authors also demonstrate that Enpp1 can act in areas that are remote from its expression site and that ectopic mineralization of soft tissue results in the accumulation of cells displaying osteoclastic features, suggesting that osteoclasts might promote regression at these sites.

**Implications and future directions**

Even though the chemical mechanism by which ENPP1 regulates biomineralization is relatively well understood, there are still no established treatments for GACI. Here, the authors demonstrate the utility of zebrafish for comparative drug screening for GACI, with a proof-of-principle experiment using etidronate. Their investigation of the zebrafish *dgf* mutant suggests that osteoclast-like cells are present at sites of ectopic mineralization, a phenomenon that has so far not been investigated in mammalian systems in any detail. The finding could have important implications for the treatment of human GACI: bisphosphonates might inhibit further mineralization but could also elicit an undesirable side effect by inhibiting the regression of soft tissue calcifications through inhibition of osteoclast function. The use of transgenic zebrafish reporter lines and *in vivo* imaging will permit a better understanding of the cellular response to ectopic mineralization in soft tissues.

## RESULTS

### *dgf*^hu4581^ mutants show decreased phosphodiesterase activity and multiple ectopic calcifications

**Fig. 1.**
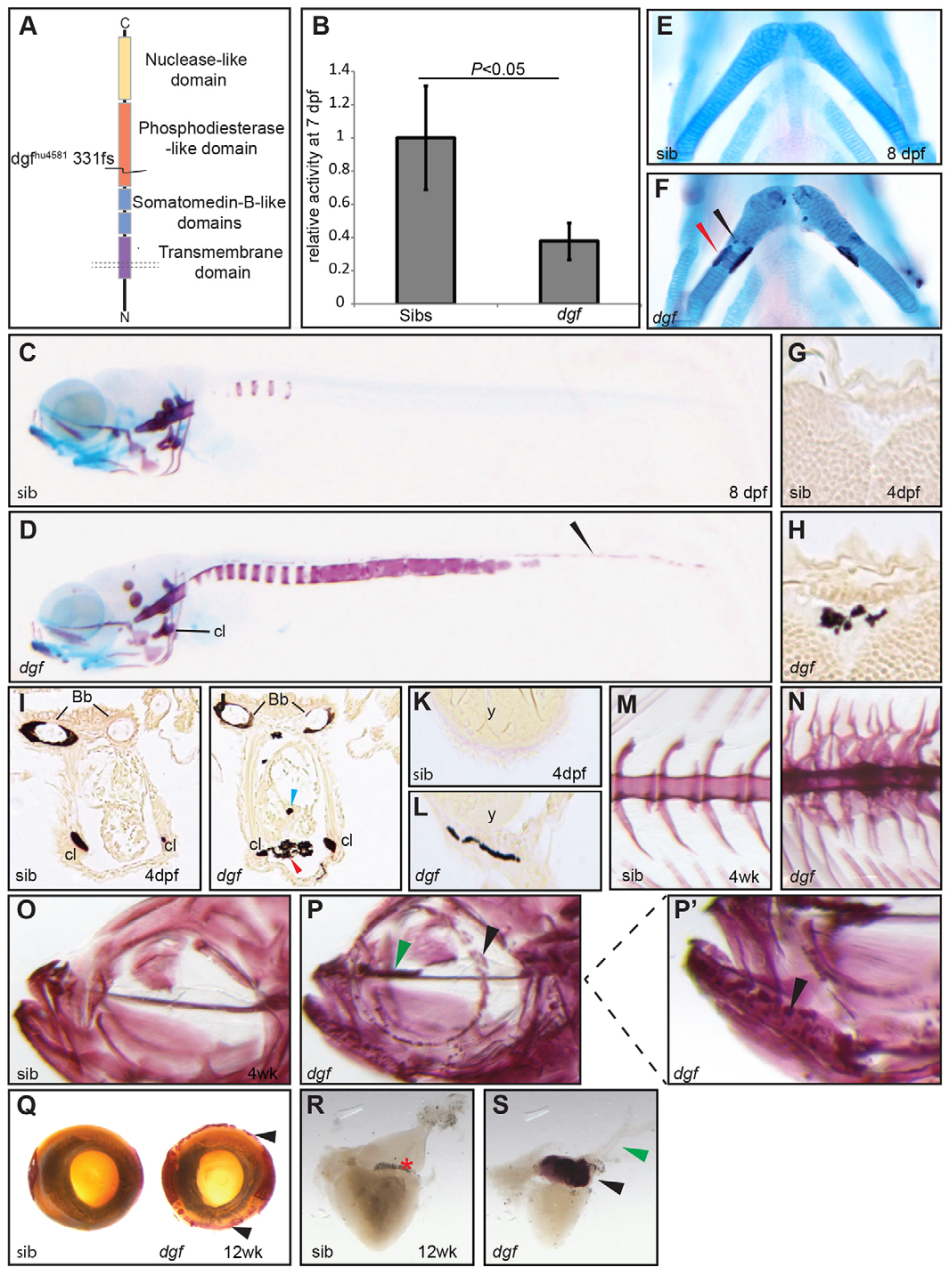
***dgf*^hu4581^ mutants show decreased phosphodiesterase activity and multiple ectopic calcifications.** (A) Depiction of the Enpp1 protein structure; the *dgf*^hu4581^ allele represents a frame shift at amino acid 331, leading to a premature stop codon. (B) Phosphodiesterase I activity is significantly reduced in the lysate of *dgf* embryos. Means±1 s.d. are shown. Sib(s), wild-type sibling(s). Alizarin-Red (staining mineralized tissue) and Alcian-Blue (staining cartilage) staining of a sibling embryo (C) and *dgf* mutant (D) at 8 dpf showing extensive ectopic calcification of the notochord, as well as calcification of the neural tube (D, arrowhead). (E) Ventral view of ceratohyal cartilage element of sibling embryo; in mutant embryos, early onset of perichondral ossification (F, red arrowhead), as well as spots of ectopic cartilage calcification (F, black arrowhead), were observed. van Kossa (brown, staining mineralized tissue) and van Gieson (red, staining osteoid) staining on transverse sections of the brain of a sibling (G) and a *dgf* mutant with intracranial calcification (H). Transverse section through the heart region of sibling (I) and mutant (J) embryos, both displaying mineralized cleithra (cl) and basobranchial (bb). Mutants (J) in addition display ectopic mineralization between myocard and epicard (red arrowhead) and within the heart (blue arrowhead). (K) Transverse section at the level of the yolk sac of a sibling; (L) the mutant displays ectopic mineralization of the skin. Axial skeleton at the level of the dorsal fin of a sibling (M) and mutant (N) 4-week-old (4wk) fish. Mutants display not only fusion of vertebral bodies but also of neural and haemal arches (N). Alizarin-Red staining of juvenile sibling (O) and mutant (P and enlarged image of the indicated area in P′). Note the ectopic mineralization at the ethmoid plate cartilage element (green arrowhead in P) and nodules of mineralization at the dentary (black arrowheads in P,P′). (Q) Alizarin-Red staining showing ectopic mineralization (black arrowheads) surrounding the eye of a *dgf* adult mutant (also green arrowhead in P). (R) In the heart of adult zebrafish, no mineralization was visible in siblings. (S) In mutants extensive ectopic calcification was found upon Alizarin-Red staining in the bulbus arteriosus (black arrowhead) but not in the ventral aorta (green arrowhead). Bb, basobranchial; Cl, cleithrum; y, yolk.

In a forward genetic screen, we have previously identified a mutant that exhibited distinct patterns of increased mineralization; furthermore, we described the positional cloning and molecular characterization of this allele, called *dgf*^hu4581^ (Huitema et al., 2012). The allele harbors a splice acceptor mutation leading to a predicted frame shift and a subsequent early stop codon within the phosphodiesterase-like catalytic domain of Enpp1 (Fig. 1A). Here, to evaluate the contribution of Enpp1 towards overall phosphodiesterase activity, we performed phosphodiesterase measurements on the lysates of *dgf*-mutant embryos. The results showed a reduction of phosphodiesterase activity by over 60%, indicating that Enpp1 accounts for the majority of phosphodiesterase activity in zebrafish embryos at this stage (Fig. 1B).

Histological examination by Alcian Blue and Alizarin Red, and van Kossa and van Gieson staining revealed multiple sites of ectopic mineralizations in *dgf* embryos. The most prominent phenotypic consequence of the *dgf* mutation was the mineralization of the notochord sheet (Fig. 1C,D; supplementary material Fig. S1A,B), which becomes apparent in all *dgf*-mutant embryos, to a variable degree, between 6 and 9 days post-fertilization (dpf). We also observed ectopic mineralization of the neural tube (Fig. 1C,D; supplementary material Fig. S1C,D), on the ceratohyal cartilage (Fig. 1E,F) and on cartilage elements of the pectoral fin (supplementary material Fig. S1E,F). Furthermore, we observed early onset of perichondral ossification in 90% of the mutants (*n*=20) (Fig. 1E,F). The first manifestation of the phenotype was detectable at 4 dpf in some *dgf* mutants. At this stage, the embryos showed calcifications in the inter-cranial space (Fig. 1G,H; supplementary material Fig. S1G,H) and within the myocardium (Fig. 1I,J), as well as calcifications in the area surrounding the myocardium (Fig. 1I,J, red arrowhead) and in the skin beneath the yolk sac and heart (Fig. 1K,L; supplementary material Fig. S1G,H). Occasionally, mutants survived to juvenile and young-adulthood stages; however, these showed reduced growth compared with their wild-type siblings (supplementary material Fig. S1I) and fusion in their axial skeleton, not only of vertebral bodies but also of neural and haemal arches (Fig. 1M,N). Furthermore, we saw ectopic calcifications in the eye (Fig. 1O,P,Q) and the ethmoid plate cartilage (Fig. 1O,P), as well as patchy mineralization of craniofacial bone elements (Fig. 1O–P′) and mineralization of the bulbus arteriosus – the outflow tract of the heart (Fig. 1R,S; supplementary material Fig. S1J,K).

In summary, *dgf* mutants display ectopic mineralizations in a number of different soft tissues, with some variability depending on developmental timing and the site of mineralization. Ectopic mineralizations of the notochord sheath and of the pectoral fin cartilage were found relatively consistently, whereas ectopic mineralization in other tissues, such as the skin and the heart, demonstrated a higher degree of variation between clutches. Part of this variation can probably be attributed to genetic variation because zebrafish, in contrast with mice, are not maintained as inbred lines.

### Soft tissue calcifications in *dgf* mutants probably represent passive calcium depositions

**Fig. 2.**
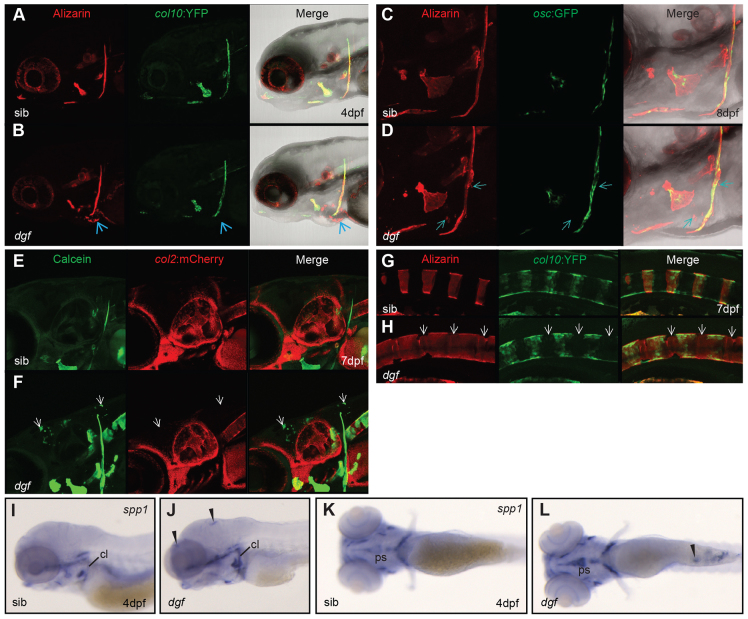
**Soft tissue calcifications in *dgf* mutants probably represent passive calcium depositions.** *collagen10a1*:YFP (col10) transgene in sibling (A) and mutant (B). Note that no expression of *collagen10a1* was detected at sites of ectopic mineralization (Alizarin Red) at the heart (B, blue arrow). *osteocalcin*:GFP (*osc*) combined with Alizarin staining in siblings (C) and *dgf* mutants (D), no ectopic expression of *osc* was observed to colocalize with ectopic mineralization in the heart region and pectoral fin (D, blue arrows). Calcein staining marks calcifications in *collagen2a1a*:mCherry (*col2*) transgenic line in wild-type siblings (E) and *dgf* mutants (F). The *dgf* mutant shows ectopic calcifications in the cranium (F, white arrows), however no ectopic expression of *collagen2a1a* was observed. Alizarin staining and *collagen10a1*:YFP transgene expression in the axial skeleton of a sibling (G) and *dgf* embryo (H), ectopic mineralization of the notochord sheet occurs independently of *collagen10a1* expression (white arrows, H). *In situ* hybridization for *spp1* (blue) in siblings (I,K) and dgf mutants (J,L). Note upregulation of *spp1* in mutant bone elements. Further ectopic expression occurs at loci that are frequently affected by ectopic mineralization in mutants (arrowheads in J,L; compare with supplementary material Fig. S1H). cl, cleithrum; ps, parasphenoid; sib, sibling.

Arterial calcification, a particularly well-studied form of soft tissue calcification, has been shown to be associated with the ectopic expression of bone and cartilage markers in mouse and human, respectively (Johnson et al., 2005; Neven et al., 2007). This has been investigated less intensively in other tissues. To examine whether the *dgf* phenotype is caused by ectopic differentiation of bone or cartilage cells, we used transgenic reporter lines for the cartilage marker *collagen2a1a* (Mitchell et al., 2013), as well as for *collagen10a1* (Mitchell et al., 2013) and *osteocalcin* (Vanoevelen et al., 2011); the latter two represent osteoblast markers in zebrafish. Importantly, these genes have been shown to be expressed in calcified arteries of *Enpp1*-knockout mice (Johnson et al., 2005). In brief, we could not detect any ectopic expression of these markers at the loci of ectopic calcifications in *dgf* mutants. Examples are shown for *collagen10a1* (Fig. 2A,B) and *osteocalcin* in the heart region (Fig. 2C,D), as well as for *collagen2a1a* in the cranium (Fig. 2E,F). Within the axial skeleton of wild-type embryos, *collagen10a1* expression colocalized with mineralized vertebral bodies (Fig. 2G). Ectopically mineralized areas of the notochord, however, were devoid of *collagen10a1* expression (Fig. 2H).

Although we were unable to detect changes in any of the above markers for chondrocytes or osteoblasts, we did see changes in the appearance of *secreted phosphoprotein 1* (*spp1*; also known as Osteopontin), a calcium-binding regulator of mineralization, which is regularly detected in conjunction with ectopic calcifications (Giachelli and Steitz, 2000). Performing *in situ* hybridizations, we detected a pattern of *spp1* expression in *dgf* mutants, which correlated with loci that often develop ectopic mineralizations, such as the cranium and the skin covering the yolk sack (Fig. 2I–L, compare supplementary material Fig. S1J,K). Moreover, *spp1* showed increased expression in the skeletal elements of *dgf* embryos when compared with siblings (Fig. 2I–L).

Spp1 is known to be expressed by mature osteoblasts, but inflammatory cells and osteoclasts are also known to express high levels of *spp1* (Sodek et al., 2000). Given the absence of other typical osteoblast or cartilage markers at loci of ectopic calcifications, it is probable that the ectopic mineralizations we find in mutants are formed in a passive process that does not involve any osteogenic cell fate change and that expression of *spp1* is a consequence of ectopic calcification.

### Treatment with the bisphosphonate etidronate is sufficient to rescue aspects of the *dgf* phenotype

**Fig. 3.**
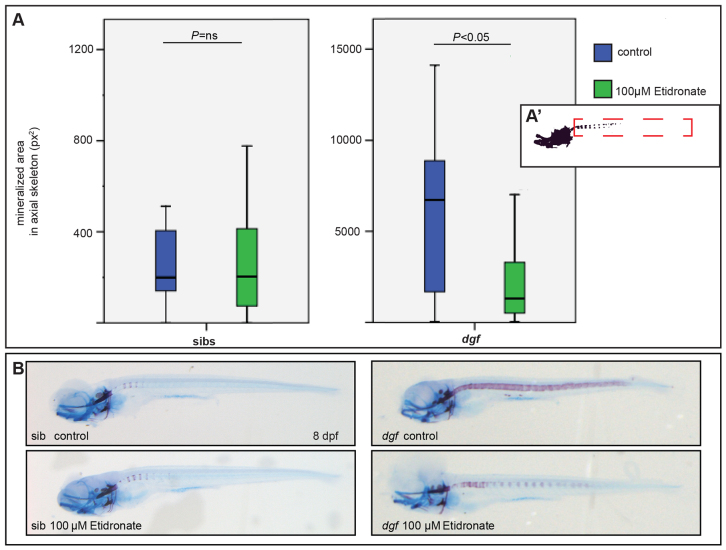
**Treatment with the pyrophosphate analog etidronate rescues aspects of the *dgf* phenotype.** (A) Measurements (in pixel area) of the mineralized area in the axial skeleton of Alizarin-Red- and Alcian-Blue-stained siblings (sibs; left panel) and mutants (right panel), which were either untreated (blue) or treated with 100 μM Etidronate (green). A′ indicates the region of interest that was measured for the analysis. No significant difference (ns) occurs in siblings (A,B left panels); in treated *dgf* mutants, the mineralized area was significantly reduced when compared with untreated *dgf* mutants (A,B right panels). *n*=50 (*dgf* 100 μM Etidronate); *n*=48 (*dgf* control); *n*= 24 (siblings 100 μM Etidronate); *n*=24 (siblings control).

We have previously shown by generation of double mutants that Enpp1 and Entpd5 are crucial proteins in establishing phosphate homeostasis in zebrafish (Huitema et al., 2012). Here, we wanted to test whether putatively reduced pyrophosphate levels in *dgf* mutants can be rescued by treatment with etidronate, a non-hydrolysable pyrophosphate analog, which has been used previously as a treatment for GACI in humans (Edouard et al., 2011). We applied the compound from 4 to 8 dpf, the time when vertebral bodies start to mineralize and when ectopic mineralizations around the notochord become apparent for the first time in *dgf* mutants. At 8 dpf, we measured the mineralized area in the notochord in treated and untreated embryos (Fig. 3A′). No significant difference was detected between the treated and untreated siblings (Fig. 3A,B), but we found a significant reduction of the mineralized area in *dgf* mutants that had been treated with 100 μM etidronate as compared with that of the untreated population (Fig. 3A,B). This experiment indirectly confirms that the phenotype of *dgf* mutants is the result of decreased pyrophosphate levels. Furthermore, it underscores the suitability of the mutant for comparative testing of compounds that have putative inhibitory effects on mineralization.

### Regulators of phosphate homeostasis and mineralization show altered expression levels in *dgf* mutants

**Fig. 4.**
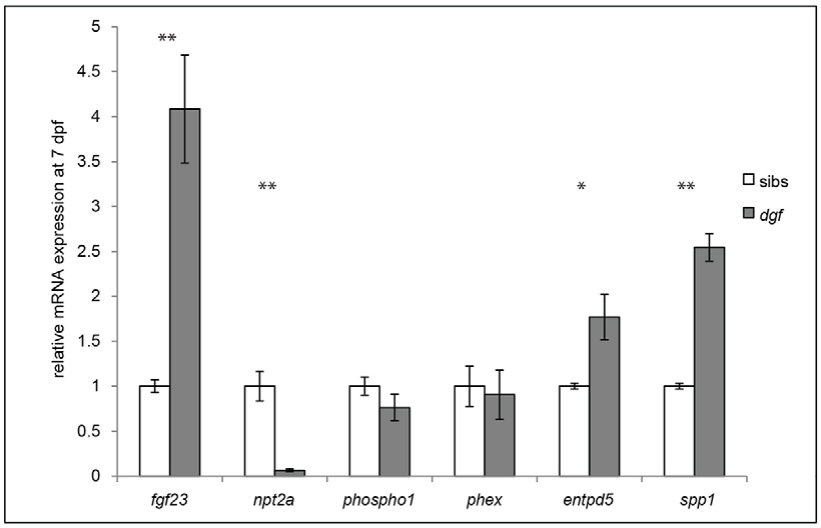
**Expression of regulators of phosphate and biomineralization is perturbed in *dgf* mutants.** qPCR analysis showing the relative gene expression levels of genes involved in phosphate homeostasis and biomineralization in siblings (sib) compared with those in *dgf* mutants, normalized to the expression of *ef2a*. *P≤0.05, **P≤0.01. Means±s.e.m. are shown.

It is known that Enpp1 loss-of-function leads to the deregulation of genes that are involved in the regulation of phosphate levels and mineralization, particularly *FGF23* (Lorenz-Depiereux et al., 2010; Mackenzie et al., 2012b) and *Spp1* (Johnson et al., 2003; Aiba et al., 2009). We performed quantitative (q)PCR analysis on RNA that had been isolated from siblings and *dgf* mutants at 7 dpf. We could indeed detect a fourfold upregulation of *fgf23* and, in concordance with this, downregulation of *npt2a*, which encodes a phosphate channel in the kidney and is negatively controlled by FGF23 (Hori et al., 2011) (Fig. 4). Furthermore, we could detect upregulation of *entpd5* and *spp1* (Fig. 4; also compare *in situ* hybridization in Fig. 2I–L), whereas *phex* and *phospho1* transcript levels remained unchanged.

### Restricted expression of *enpp1* is sufficient to rescue ectopic mineralizations in the notochord sheet

**Fig. 5.**
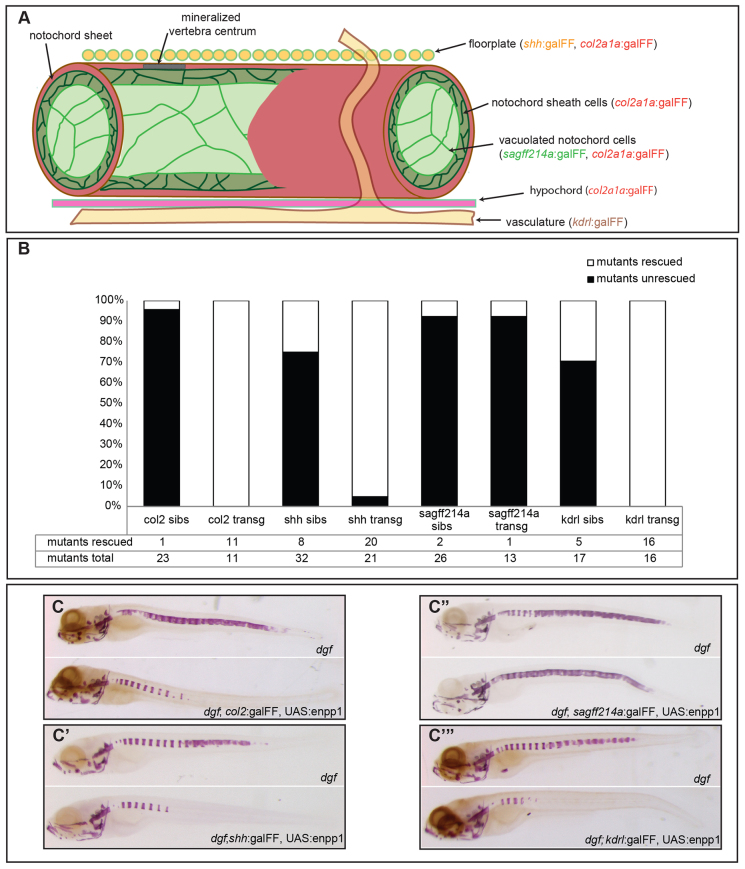
**Distal expression of *enpp1* is sufficient to prevent ectopic calcifications in the notochord.** (A) Overview of the tissue-specific lines that were used in the different rescue experiments. (B) Analysis and quantification of *dgf* embryos and *dgf* transgenic lines with respect to the notochord phenotype (shown as percentages of the total number of embryos examined). Sibs, siblings; transg, transgenic line. (C–C‴) Representative examples of non-transgenic *dgf* embryos and their transgenic *dgf* siblings for the respective constructs after Alizarin-Red staining.

In mouse, it has been shown that *Enpp1* is expressed in a wide array of tissues, and high expression levels occur in bone, liver, kidney and skin (Murshed et al., 2005). Similarly, performing *in situ* hybridization for *enpp1* on zebrafish embryos revealed a ubiquitous expression pattern, and bone elements showed pronounced levels of *enpp1* expression (supplementary material Fig. S2). To our knowledge, no *in vivo* experiments have addressed whether the ubiquitous expression of *enpp1* indicates a general requirement in tissues for the protein to prevent ectopic calcification, or whether Enpp1 acts in a spatially restricted manner. To clarify this, we employed a UAS-galFF overexpression system. For this, we generated a UAS:*enpp1*-ires-TagRFP transgenic line; functionality of the Enpp1 protein was confirmed by the phosphodiesterase assay (supplementary material Fig. S3), and expression of red fluorescent protein (RFP) allowed us to identify *enpp1*-expressing cells. Because mineralization of the notochord sheet provided a reliable readout, we combined our UAS:*enpp1*-ires-TagRFP line with galFF lines that expressed *enpp1* within, or in the vicinity of, the notochord. It has recently been shown that cells within the notochord sheet contribute to the initial mineralization of vertebral centra in teleosts (Grotmol et al., 2005; Bensimon-Brito et al., 2012; Wang et al., 2013). We therefore expected that *enpp1* expression inside the notochord, driven by a transgenic *col2a1a* promoter (*col2a1a*:galFF line; Dale and Topczewski, 2011), would be sufficient to rescue the notochord phenotype of *dgf* mutants. Because the *col2a1a* promoter is also active in vacuolated notochord cells and, to a lower level, in the floorplate and hypochord, we also made use of lines that expressed *enpp1* either exclusively in vacuolated notochord cells (*sagff214a*:galFF) (Yamamoto et al., 2010) or in the floorplate (*shh*:galFF) (Ertzer et al., 2007). In addition, we tested whether expression from blood-vessel endothelial cells (*kdrl*:galFF; formerly known as *flk1*) (Beis et al., 2005) was sufficient to rescue the notochord mineralization. Fig. 5A depicts a scheme of the lines and promoters that were used. Of note, transgenic embryos were indistinguishable in length and morphology from their non-transgenic wild-type siblings. At 9 dpf, we performed Alizarin-Red staining of bone and scored for the presence of ectopic mineralizations in the notochord sheet.

We found that expression not only from the *col2a1a* promoter (Fig. 5B,C) but also solely from blood vessels (Fig. 5B,C‴) and the floorplate (Fig. 5B,C′) was sufficient to completely prohibit ectopic mineralizations in the notochord sheet in *dgf* mutants. Given that the expression of *enpp1* in notochord sheath cells showed substantial rescue, we were surprised to find that *enpp1* expression in vacuolated notochord cells did not prohibit ectopic mineralization of the notochord sheath (Fig. 5B,C″).

These experiments indicate that *enpp1* can act at locations that are remote from its site of expression; however, it remains to be established whether a secreted form of the protein or the diffusion of pyrophosphate are the main contributors to this effect.

### Cells that express osteoclastic markers appear at ectopic mineralization sites in *dgf* mutants

The ectopic expression of *spp1* in the absence of other bone markers at sites of ectopic mineralization prompted us to address the possibility that osteoclasts or macrophages are present at those sites. It has been demonstrated that Spp1 is expressed by osteoclasts (Merry et al., 1993) and macrophages (Giachelli et al., 1998) and is of importance in the cellular response to ectopic calcifications (Steitz et al., 2002).

**Fig. 6.**
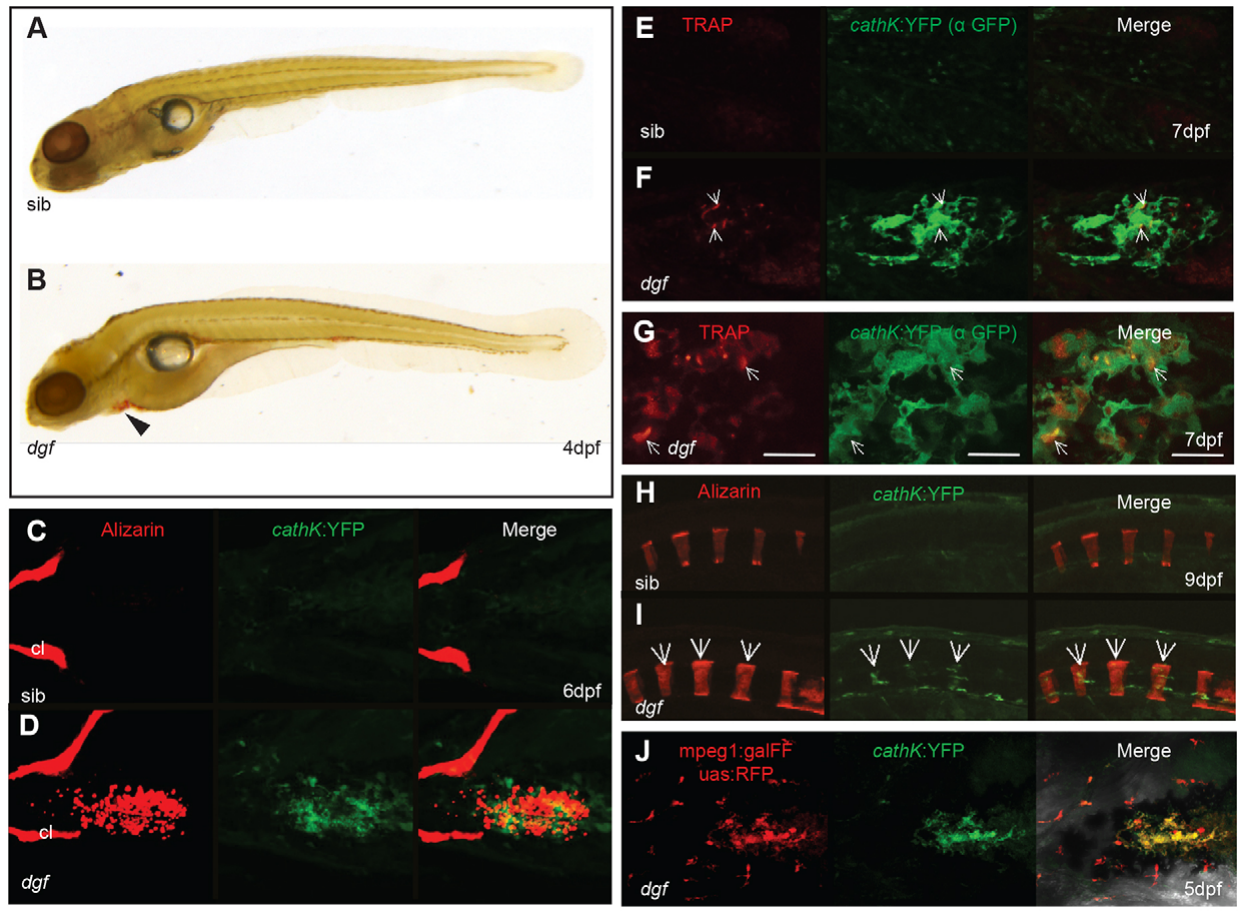
**Cells expressing osteoclastic markers appear at ectopic mineralization sites in *dgf* mutants.** Staining of Trap in a wild-type sibling (A) and *dgf* embryo (B). Trap staining (red) was visible in a *dgf* embryo at the region of the heart and yolk sac (B, arrowhead). Ventral view of sibling (C) and mutant (D) embryo with ectopic mineralization in the heart and yolk sac region. (C) No *cathepsinK*-positive cells were visible in siblings at this timepoint; (D) mutants showed colocalization of ectopic soft tissue mineralization and *cathepsinK*-positive cells, but no *cathepsinK*-positive cells were aligned to skeletal elements, such as the cleithrum (cl). (E,F) Ventral view of the yolk sac area of embryos that had been stained for Trap and with an antibody against GFP (α GFP). (F) Trap staining appeared in association with *cathepsinK*-positive cells. White arrows indicate loci of osteoclasts with high Trap activity. (G) Higher-magnification image of *cathepsinK* and Trap colocalization in a *dgf* mutant. Scale bar: 20μm. The staining of Trap in E–G is pseudo-colored for improved visibility. In the axial skeleton, *cathepsinK*-positive cells were not visible in siblings (H); however, in *dgf* mutants (I), *cathepsinK*-positive cells appeared from 9 dpf onwards and colocalized with mineralized vertebral bodies. White arrows indicate osteoclasts aligning with vertebral elements. (J) Ventral view of accumulating *cathepsinK*-positive cells in the skin of the heart region. These cells also express the macrophage marker *mpeg1*.

Staining of Tartrate resistant acid phosphatase (Trap) and the expression of *cathepsinK* have previously been shown to be suitable markers for osteoclasts in teleosts (Witten et al., 2001; Chatani et al., 2011; To et al., 2012). We therefore made use of Trap staining and a *cathepsinK*:YFP reporter line to investigate the presence of osteoclasts in *dgf* mutants. Trap-positive cells have only been reported to appear in zebrafish after 12 dpf (Hammond and Schulte-Merker, 2009). In *dgf* mutants, however, we found Trap staining as early as 4 dpf (Fig. 6A,B). This is the timepoint at which ectopic calcifications first became detectable in mutants, and Trap staining indeed appeared at loci that were associated with ectopic calcifications in embryos of this stage (compare supplementary material Fig. S1K). To establish a direct connection between ectopic calcifications and osteoclasts, we made use of the *cathepsinK*:YFP reporter (which was generated in our laboratory and is described in Bussmann and Schulte-Merker, 2011). Using this line, we confirmed that cells expressing *cathepsinK* colocalized with ectopic calcification sites in *dgf*-mutant embryos (Fig. 6C,D). We could not observe any cells that expressed high levels of *cathepsinK* or stained positive for Trap in sibling embryos (Fig. 6A–D). In contrast to the association of ectopic mineralizations and osteoclasts, we could hardly find any Trap-or *cathepsinK*-positive cells associated with skeletal elements, such as the cleithrum (Fig. 6B,D). This indicates that the expression of *spp1* in those loci is not derived from osteoclasts but probably osteoblasts, which are associated with these skeletal elements (compare Fig. 2A). To correlate Trap staining and *cathepsinK* expression, we combined Trap staining with the subsequent staining of YFP (using an antibody against green fluorescent protein) in *cathepsinK*:YFP-positive embryos. Indeed, we found Trap staining in close association with a subset of *cathepsinK*-positive cells (Fig. 6E–G). The lack of complete congruency of the staining patterns of *cathepsinK*:YFP and Trap can probably be attributed to the fact that YFP is active in the cytosol, whereas Trap accumulates in secretory compartments of the cell (Ljusberg et al., 2005). From 9 dpf onwards, we also found *cathepsinK*-positive cells appearing in association with vertebral elements in *dgf* mutants but, again, not in siblings (Fig. 6H,I). Of note, for reasons of comparability, we focused on observations in the skin of the heart and yolk sac area; we could, however, also observe soft tissue calcification, which was associated with cathepsinK-positive cells, in other loci, such as the brain and the heart itself (not shown). To further validate that these cells were osteoclasts, we combined the *cathepsinK*:YFP line with a reporter line for the macrophage marker *mpeg1* (Ellett et al., 2011) (*mpeg1*:gal4, UAS:RFP). We found that *cathepsinK*-positive cells in *dgf* mutants expressed comparable levels of RFP to macrophages, for which we could not detect any *cathepsinK*:YFP expression (Fig. 6J). The presence of this macrophage-associated marker on osteoclasts is consistent with their origin from cells of the mononuclear phagocyte lineage. Finally, we tested whether treatment with the inflammatory inhibitors ibuprofen or sulindac, between day 3 and day 6, would have an effect on the severity of the ectopic mineralization phenotype; however, we could not detect any significant results (supplementary material Fig. S4).

## DISCUSSION

Here, we provide analysis of the zebrafish mutant *dgf*, which shows ectopic mineralizations in a number of tissues. The *dgf*^hu4581^ allele represents a mutation that leads to an early stop codon within the catalytic domain of the Enpp1 protein (Huitema et al., 2012). This is reflected by a strong reduction of phosphodiesterase activity in the lysate of *dgf* mutants. Similarly, mutations in the phosphodiesterase domain, which have been detected in a number of GACI individuals, have been shown to cause loss of ENPP1 activity (Rutsch et al., 2003).

The zebrafish *dgf* phenotype shows many of the features that have been described in the clinic as a consequence of *ENPP1* mutation in the human syndromes GACI and PXE (Rutsch et al., 2003; Li et al., 2012; Nitschke et al., 2012), and that are observed in mouse upon mutation of *Enpp1* (Johnson et al., 2003; Murshed et al., 2005; Mackenzie et al., 2012b; Li et al., 2013) – most notably, ectopic calcifications in the skin and cartilaginous elements of embryos, as well as in the eye and bulbus arteriosus of juvenile to adult fish. The absence of arterial calcifications in zebrafish embryos can probably be attributed to the morphological differences in the arteries of zebrafish embryos when compared with the medium and large arteries of human and mouse, which have multiple layers of vascular smooth muscle cells. We did, however, find ectopic calcifications in the bulbus arteriosus, which is the outflow tract of the heart in juvenile and adult fish. This structure shows histological similarities to arteries, including a thick circumferential layer of smooth muscles (Hu et al., 2001). The intracranial calcifications along the midline that we observe in zebrafish embryos have, so far, not been reported in individuals with *ENPP1* mutations; however, an individual suffering from hypophosphatasia due to a *KLOTHO* mutation has been diagnosed with calcifications along the midline of the brain (Ichikawa et al., 2007).

Arterial calcification due to loss of ENPP1 function has been demonstrated to be associated with the expression of bone and cartilage markers in *Enpp1*^−/−^ mice (Johnson et al., 2005). By contrast, no expression of bone markers has been observed in the calcified arteries of *Mgp* knockout mice (Luo et al., 1997). Using transgenic marker lines, which allow *in vivo* expression analysis at high resolution, we could not observe such events in any of the ectopically mineralized tissues of *dgf*-mutant embryos. This is in line with findings by Murshed et al. (Murshed et al., 2005) who postulated that the presence of fibrillar collagen and the removal or absence of pyrophosphate are sufficient for the occurrence of calcifications (Murshed et al., 2005). Indeed, a number of tissues where we observed ectopic calcifications, such as the skin (Le Guellec et al., 2004), cartilage elements and the notochord (Fang et al., 2010; Dale and Topczewski, 2011; Mitchell et al., 2013), as well as the bulbus arteriosus (Hu et al., 2001), are known to be rich in fibrillar collagen. Although we cannot exclude that cellular changes at the level of matrix vesicles play a role in the mineral nucleation at those loci, it is probable that the reduced levels of pyrophosphate and the presence of fibrillar collagen are the main determinants for the induction of ectopic mineralizations. In conclusion, we believe that the correlation of ectopic calcifications with the ectopic appearance of bone and/or cartilage markers might be a common event in the (mammalian) vasculature, but not necessarily other tissues. In line with these findings, Murshed et al. have also reported the absence of osteoblast markers upon the induction of calcification in the dermis of mice (Murshed et al., 2005).

The ability of the bisphosphonate etidronate to rescue the notochord mineralization of *dgf* mutants not only further supports the notion that loss of pyrophosphate is likely to be the determining factor for the *dgf* phenotype but also demonstrates the suitability of this zebrafish model to screen and evaluate other mineralization inhibitors, such as other bisphosphonates or thiosulfate.

Human genetic studies linking *ENPP1* mutations to increased FGF23 levels and subsequent hypophosphatemia (Lorenz-Depiereux et al., 2010) have recently been confirmed in an *Enpp1^−/−^* mouse model (Mackenzie et al., 2012b). In zebrafish, we observed a similar situation with strong upregulation of *fgf23* and downregulation of *npt2a*, a transporter responsible for phosphate resorption in the kidney under the control of *fgf23* (Hori et al., 2011). By contrast, we could not find differential expression of *phex1*, a regulator of *fgf23* (Rowe, 2012).

Although we cannot provide direct proof in the form of serum phosphate levels, which is a limitation inherent to the zebrafish model, it is likely that *dgf* mutants are hypophosphatemic, a notion that is supported by upregulation of *entpd5*, which we believe complements alkaline phosphatase as a local source of phosphate in the microenvironment of osteoblasts in zebrafish (Huitema et al., 2012). Upregulation of *spp1* in bone elements of *dgf* zebrafish is contradictory to observations that have been made in osteoblast cultures derived from *Enpp1* knockout mice, where *Spp1* expression was decreased (Johnson et al., 2003). Conversely, *Spp1* has been reported to be upregulated in spinal hyperostosis of *twy* mice, which represent another *Enpp1* allele (Aiba et al., 2009). Mechanistically, the upregulation of *spp1* in mutants could derive from the premature maturation of osteoblasts or a negative feedback response.

Using a UAS and galFF-based approach, we provide evidence that *enpp1* expression is crucial in the vicinity of sites of ectopic mineralizations, but does not need to be expressed in the affected tissue directly, as demonstrated by the rescue of ectopic notochord mineralization by the expression of *enpp1* in the floorplate or blood vessels. It is possible that although *enpp1* is widely expressed, it does not need to be provided locally, but can act across tissue boundaries because pyrophosphate and/or a secreted form of Enpp1 can act at loci remote from their site of expression. These factors must be readily diffusible *in vivo*, which is particularly evident from the observation that expression from the floorplate, a single line of cells dorsal to the notochord, is sufficient to completely rescue ectopic mineralizations of the notochord in embryos at 9 dpf. Surprisingly, expression from vacuolated notochord cells alone was not sufficient to rescue the phenotype. This might be explained by the epithelial nature of notochord sheath cells (Dale and Topczewski, 2011), which enclose the vacuolated notochord cells and probably function as a diffusion barrier.

Additionally, we show that early zebrafish embryos do have the potential to generate cells that express the typical osteoclast markers Trap and *cathepsinK*, which are normally only found much later in development (Hammond and Schulte-Merker, 2009). These cells colocalize with ectopic mineralizations in *dgf* embryos and represent a subpopulation of cells that express the macrophage marker *mpeg1*, indicating they are derived from the monocyte-macrophage lineage. The combination of these features strongly suggests that these cells represent a type of osteoclast that develops as a response to ectopic calcifications. In a few other instances, it has been shown previously that ectopic bone and hydroxyapatite fragments can induce osteoclast-like multinucleated giant cells (Krukowski and Kahn, 1982). Arterial calcification has previously been associated with the presence of osteoclasts (Min et al., 2000) and, more recently, Bas and colleagues have suggested an active process of mineral resorption that is mediated by CD68^+^ cells in a rat model for medial artery calcification (Bas et al., 2006). Furthermore, osteoclast-like cells have been described to be associated with calcified atherosclerotic plaques (Jeziorska et al., 1998; Doherty et al., 2002). Although it is difficult to compare these findings directly, we believe it will be important to consider the existence of these soft tissue calcification-associated osteoclasts in humans because they could have important consequences for the treatment of GACI. Bisphosphonates, which are currently being used in the treatment of GACI (Rutsch et al., 2008), might not only prevent further progression of calcifications but, at the same time, also hinder their regression because bisphosphonates are widely known for their capacity to inhibit osteoclast function.

Here, we introduced the *dgf* zebrafish mutant, which represents a valuable model for investigating Enpp1 function and ectopic mineralization, and extend, through the present analysis, our understanding of Enpp1 function *in vivo. dgf* mutants show a number of features that are also found in GACI and PXE individuals with ENPP1 mutations; most importantly, mineralization in cartilage elements, skin and the circulatory system. Our data underline the crucial function of phosphate and pyrophosphate homeostasis in the regulation of biomineralization across species, and we demonstrate the potential of Enpp1 to exert its function across tissues. Lastly, we show that ectopic mineralizations in soft tissue lead to a rapid osteoclastic cellular response, something which has not been fully explored in a murine or human setting.

## MATERIALS AND METHODS

### Zebrafish maintenance

Fish were maintained and raised under standard husbandry conditions (Brand, 2002) and according to Dutch guidelines for the care and housing of laboratory animals.

### Phosphodiesterase assay

Mutants and siblings (*n*=10) were sorted based on Alizarin live-staining (see below) or transgene expression. Embryos were sonicated to a homogenous suspension in purified water. The protein concentration was measured (by using a Thermo Scientific Pierce BCA Protein Assay Kit) and diluted to 600 μg/ml. The phosphodiesterase assay was scaled down to a microplate reader format (Biochrom Asys Expert 96) but essentially performed as described previously (Hynie et al., 1975). Results are shown for three independent biological replicates.

### Skeletal stainings

Alcian-Blue and Alizarin-Red staining (Walker and Kimmel, 2007) and *in vivo* skeletal staining (Spoorendonk et al., 2008) were performed with minor modifications as previously described.

For sectioning, embryos or juvenile fish were embedded in plastic and sections at 6 μm were cut on a microtome. van Kossa (Bancroft, 1996b) and van Gieson staining (Bancroft, 1996a) was performed as described elsewhere.

### Trap staining

Trap staining on zebrafish embryos was performed as described previously (Witten et al., 1997; Edsall and Franz-Odendaal, 2010). Briefly, embryos were fixed in 4% paraformaldehyde, washed in H_2_0 and incubated for 2 hours at room temperature in tartrate buffer [0.2 M acetate buffer (pH 5.5) with 50 mM sodium tartrate dibasic dehydrate]. Embryos were then incubated in Trap staining solution {6% substrate solution [2 mM naphthol-AS-TR-phosphate (N6000 Sigma) in N,N-dimethylformamide], 90.89% 0.2 M acetate buffer with 100 mM tartaric acid, 3% hexazotized pararosaniline (P3750 Sigma) and 0.01% of 0.1 M MgCl_2_} for 2 hours.

### Imaging

*In situ* hybridization and whole-mount bone staining was imaged on an Olympus SZX 16 microscope. Sections were imaged on a Zeiss Axioplan microscope. For laser confocal imaging, embryos were embedded in 0.5% low-melting-point agarose and, where applicable, anesthetized with 1.5% Tricaine mesylate. Confocal imaging was performed on a Leica SPE live-cell imaging confocal microscope using ×10 and ×20 objectives. Images were analyzed by using Leica LAS AF lite software.

### *In situ* hybridization and immunohistochemistry

*In situ* hybridization and immunohistochemistry were performed as described previously (Schulte-Merker, 2002). Templates for *in vitro* transcription of *enpp1* and *spp1* were generated from cDNA. For *enpp1*, a combination of two probes was used for improved detection. Primer sequences are shown in supplementary material Table S1. Antisense digoxygenin-labeled mRNA probes were generated according to standard protocol (Promega SP6, RNA polymerase). Digoxygenin was purchased from Roche.

For detection of YFP on embryos that had been stained for Trap, a rabbit antibody against GFP (1:300, Torrey Pines TP401) and an Alexa-Fluor-488-conjugated antibody against rabbit IgG were used (1:500, Molecular Probes A11034). Embryos were fixed in 4% paraformaldehyde and permeabilized with proteinase K (Promega, 15 μg/ml) for 3 minutes.

### Treatment with etidronate

Etidronate (Sigma-Aldrich, P5248) was dissolved in E3 medium (Brand, 2002). Embryos were placed into 100 μM etidronate in E3 medium at 4 dpf. The control group was placed in fresh E3 medium. At 8 dpf, embryos were analyzed by skeletal staining, as described above. Embryos were imaged and the mineralized area in the axial skeleton was measured by using ImageJ. Box plots summarize the results of three independent biological replicates. Groups were compared by using Student’s *t*-test.

### qPCR

Siblings were separated from *dgf* mutants at 7 dpf by using Alizarin live-staining. RNA was isolated by using the Qiagen RNeasy Kit, and a DNaseI (Promega) digest was performed on the column. RNA quality was checked on an agarose gel and measured with a Nanodrop photospectrometer (Thermo Scientific). Random hexamers were used for reverse transcription (M-MLV reverse transcriptase, Promega). The Primer 3 program was used for primer design. The primers spanned at least one intron to avoid amplification from genomic DNA. Melting temperatures and PCR efficiency were tested, and qPCR was performed using the Bio-Rad MyIQ single-color real-time PCR detection system and software. Reactions contained 12.5 μl Sybr Green (Bio-Rad), 3 μl primer mix (at 1.5 μM), 5 μl cDNA at 10 ng/μl, and 4.5 μl Millipore water. qPCR program: 3 minutes at 95°C, 10 seconds at 95°C followed by 45 seconds at the optimal primer temperature (40 cycles); 1 minute at 95°C and 1 minute at 65°C. cDNA was analyzed from three pooled clutches of embryos for the siblings and mutants. *ef1a* was used as an internal control. Groups were compared by Student’s *t*-test.

### Treatment with inflammatory inhibitors

Sulindac (Santa Cruz, sc-202823) and ibuprofen (Sigma-Aldrich, I4883) were dissolved in dimethylsulfoxide. From 3 dpf to 6 dpf, embryos were raised in E3 medium with DMSO only or E3 medium with either 5 μM ibuprofen or 10 μM sulindac. These compounds and concentrations have been shown previously to effectively inhibit inflammation in zebrafish embryos (d’Alencon et al., 2010).

### Transgenic lines

The transgenic lines used are described in brief. Details are available upon request.

#### col2a1a:galFF

We recapitulated cloning of the R2 *collagen type 2a1a* enhancer element and R3 (−116 bp) promoter region, as described previously (Dale and Topczewski, 2011), and the enhancer element was placed upstream of a galFF element (Asakawa et al., 2008) in a miniTol2 vector (Balciunas et al., 2006).

#### *shh*:galFF

The sonic hedgehog (*shh*) floorplate-specific promoter construct ar-B has been described previously (Ertzer et al., 2007), and has been cloned into a miniTol2 vector (Gordon et al., 2013). A galFF element (Asakawa et al., 2008) was inserted into this vector.

#### sagff214a:galFF

*sagff214a* and its specific expression in vacuolated notochord cells has been described previously (Yamamoto et al., 2010). The line was kindly provided by the laboratory of Koichi Kawakami (National Institute of Genetics, Shizuoka, Japan).

#### *kdrl*:galFF

A galFF element was cloned in front of a 7 kb *kdrl* promoter fragment (Beis et al., 2005) within a miniTol2 vector.

#### UAS:*enpp1*-ires-tagRFP *cmlc2*-CFP

A cDNA clone of *enpp1* was generated by using PCR and then sequenced and placed downstream of a 5×UAS element within a Tol2 vector, described previously (Asakawa et al., 2008). An ires-tagRFP element was placed downstream of *enpp1* to enable the assessment of expression levels. Further, a *cmlc2* (Huang et al., 2003) mTurquoise (cyan fluorescent protein) cassette was included for selection purposes.

#### cathepsinK:YFP

*cathepsinK*:YFP was generated previously using a BAC CH73-114M20 (Bussmann and Schulte-Merker, 2011).

#### osteocalcin:GFP, col2a1a:mCherry, col10a1:YFP, mpeg1:gal4

*osteocalcin*:GFP (*osc*:GFP) (Vanoevelen et al., 2011), *col2a1a*BAC:mcherry (*col2*:mCherry) (Mitchell et al., 2013), *col10a1*BAC:mCitrine (*col10*:YFP) (Mitchell et al., 2013), *mpeg1*:gal4 (Ellett et al., 2011) have been described elsewhere.

## Supplementary Material

Supplementary Material
