# Supplementary material for: Zebrafish enpp1 mutants exhibit pathological mineralization, mimicking features of generalized arterial calcification of infancy (GACI) and pseudoxanthoma elasticum (PXE)
Source: Dis Model Mech. 2014 Jun 6;7(7):811–22. doi: 10.1242/dmm.015693 (PMC4073271; doi:10.1242/dmm.015693)
Supplement: Supplementary Material [file supp_7_7_811__index.html]

Zebrafish enpp1 mutants exhibit pathological mineralization, mimicking features of generalized arterial calcification of infancy (GACI) and pseudoxanthoma elasticum (PXE) — Supplementary Material 

# Zebrafish *enpp1* mutants exhibit pathological mineralization, mimicking features of generalized arterial calcification of infancy (GACI) and pseudoxanthoma elasticum (PXE)

## DMM015693 Supplementary Material

**Files in this Data Supplement:**

- **Supplementary Material**
