# Supplementary material for: Zebrafish enpp1 mutants exhibit pathological mineralization, mimicking features of generalized arterial calcification of infancy (GACI) and pseudoxanthoma elasticum (PXE)
Source: Dis Model Mech. 2014 Jun 6;7(7):811–22. doi: 10.1242/dmm.015693 (PMC4073271; doi:10.1242/dmm.015693)
Supplement: Supplementary Material [file supp_7.7.811_DMM015693.pdf]

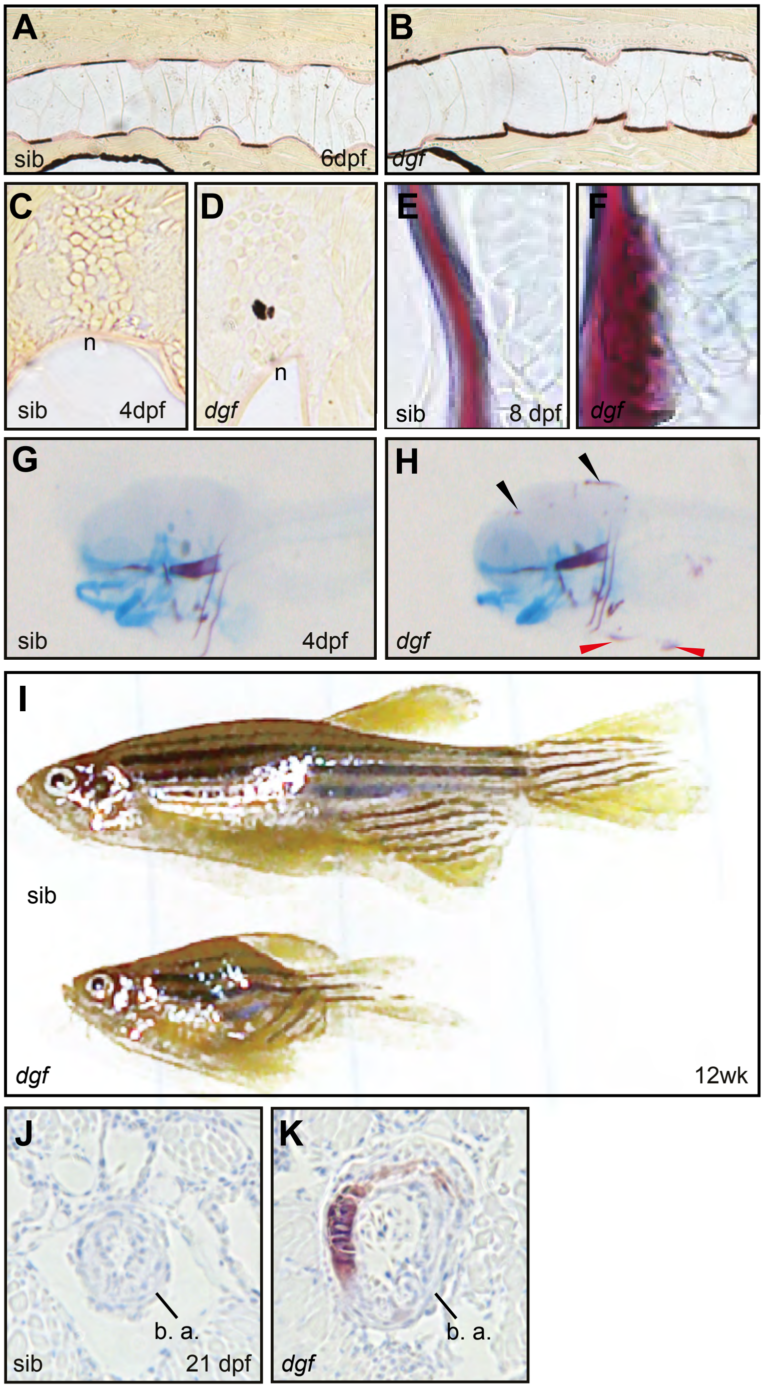

Supplemental Fig. 1. Van kossa/van Gieson staining shows segmented mineralization of the notochord sheath in siblings (A), in *dgf* mutants show ectopic calcification of the intervertebral spaces (B). Transverse section through the neuraltube of sibling (C) and mutant with ectopic calcification (D). Alizarin red/Alcian blue stained cleithrum and pectoral fin cartilage of siblings (E) and *dgf* mutant (D) showing ectopic calcification. Overview of Alizarin red/Alcian blue stained sibling (G) and mutant (H). The black arrowheads indicate cranial calcifications, the red arrowheads point at mineralizations of the skin surrounding the yolk sac and heart (H). *dgf* mutants can reach adulthood in rare cases but remain smaller (I). Transverse section of alizarin stained juvenile embryos at the bulbus arteriosos (b. a.), no mineralization is visible in silbing (J), circumferential calcification in the *dgf* mutant (K).

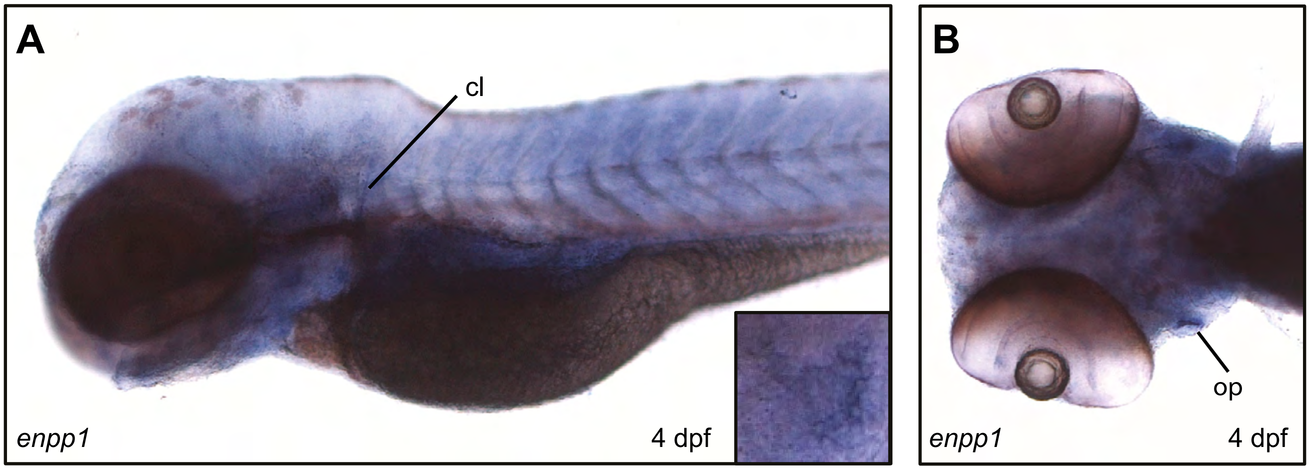

Supplemental Fig. 2. Lateral (A) and ventral (B) view of in-situ hybridisation showing the expression pattern of enpp1 at 4 dpf. Note: expression in bone elements such as cleithrum (cl) (A) and the opercle (op) (B, box in A).

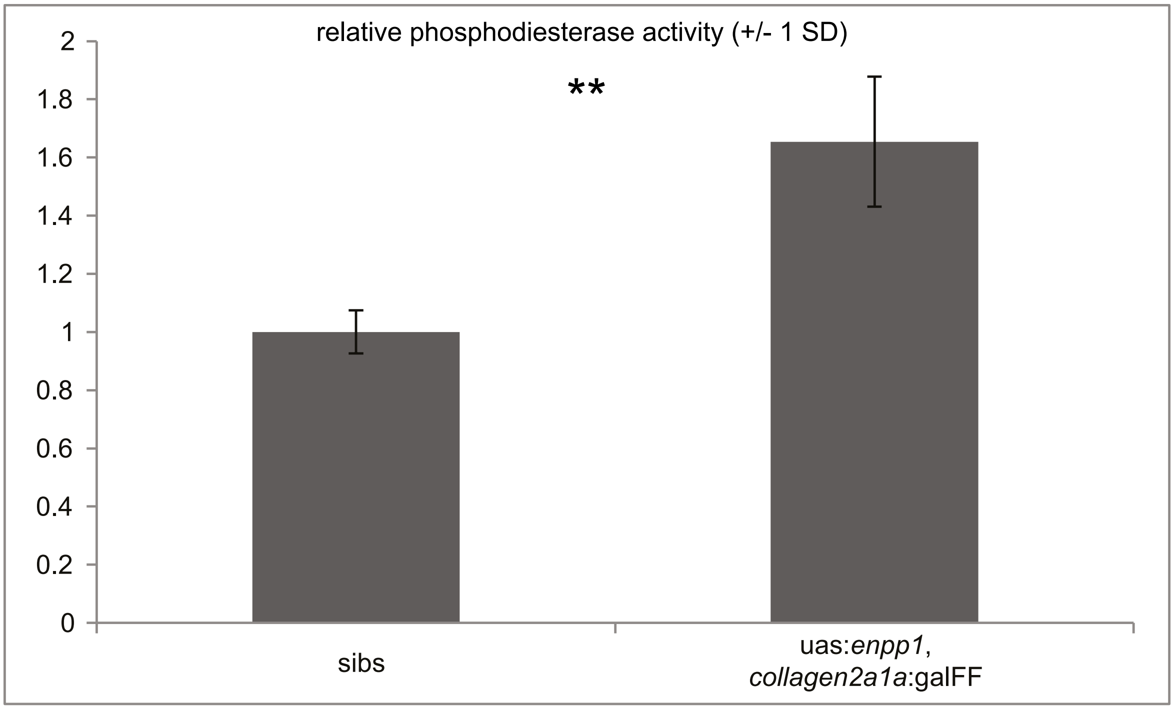

Supplemental Fig. 3. Phosphodiesterase activity is elevated in uas:*enpp1*, *collagen2a1a*:galFF embryos.

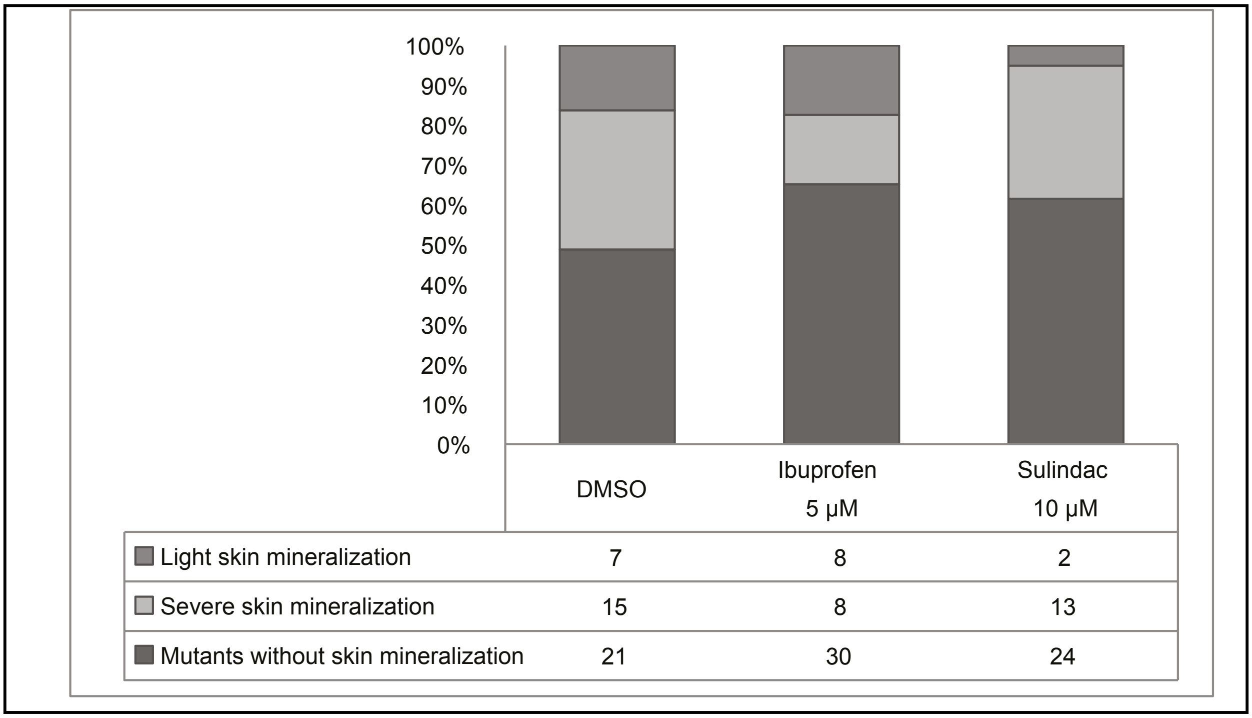

Supplemental Fig. 4. Quantification of skin mineralizations in *dgf* mutants upon treatment with inflammatory inhibitors. Numbers summarize 4 biological replicates. No significance difference between the groups could be detected using a 3x3 Chi-squared test at α=0,05.

Supplementary table 1. Primers used in *in situ hybridization* and qPCR analyses

**Gene**	**Forward (3'-5')**	**Reverse (3'-5')**
entpd5 (qPCR)	ATATGCCTGAAAAGGGTGGA	TACTTCTTTGACCTCATTCAGCAG
fgf23 (qPCR)	CGGGGCTCATACAGTGTAATC	TCCAACAGTTTGTGGTGGAA
spp1 (qPCR)	TGTGAAGCGCTCAGCAAG	CATCTGCCTCCTCAGTGTCA
npt2a (qPCR)	CAACACAGATTTCCCGTATCC	GCGGGCAGCTTCTCTTTG
ef1a (qPCR)	CTTCTCAGGCTGACTGTGC	CCGCTAGCATTACCCTCC
phex (qPCR)	CCGTCATCACGGTATCACAA	TCTGAGCCATGGGTAAATCC
phospho1 (qPCR)	TGAAAACAGGAGCAGCTGTAAA	GGGGCTGGAGATCTGCTT
spp1 (ish)	GGACCAGGCAGCTACAGAAG	CACTGCCGTCTGTCGTCTAA
enpp1-1 (ish)	AAGAAGCGGCACTTTTAGCA	ACCAGAAGGGGCAGAAAAAC
enpp1-2 (ish)	ACCTACTGGGCACAGGACAG	CACAGTCCTCTTGAAGCCAAC

qPCR, quantitative PCR; ish, in situ hybridization.
